# Glenoid Morphology and Related Parameters in Turkish Society

**DOI:** 10.7759/cureus.27959

**Published:** 2022-08-13

**Authors:** Gökhan Karademir, Ömer Aslan

**Affiliations:** 1 Department of Orthopedics and Traumatology, Acibadem University, Istanbul, TUR; 2 Department of Orthopedics and Traumatology, Avcılar Murat Kölük State Hospital, Istanbul, TUR

**Keywords:** computed tomography, related parameters, gender, ethnicity, glenoid morphology

## Abstract

Introduction

Glenoid morphology may vary in different ethnic groups. Detection of these differences may be important in preoperative planning, especially in reverse shoulder arthroplasty. In this study, we investigated the mean glenoid size and retroversion in Turkish society and their relationship with the dominant side, gender, height, weight, and body mass index (BMI).

Materials and methods

Between 2019 and 2021, 102 shoulders of 51 patients (24 females/27 males, 51 left/51 right) who were included in shoulder joint imaging during thorax CT scanning were examined. Those with glenoid fracture, arthrosis, shoulder surgery or deformity, or younger than 18 years of age were not included in the study. The mean age was 41.69 (range: 18-73) years. Glenoid anterior-posterior diameter (D) and glenoid version (GV) were measured in axial slices, and glenoid height (H) was measured in coronal slices. The correlation of these parameters with gender, height, weight, and dominant side was examined.

Results

Mean D was 25.79±4.44 mm, mean H was 29.08±4.08 mm, and mean GV was -0.99°±0.92°. The mean height of the patients was 162±16.23 cm and the mean weight was 71.9±15.36 kg. The glenoid diameter and height were smaller in females, however, no statistically significant difference was found in the glenoid version (p<0.01, p<0.01, and p=0.92). The glenoid on the dominant side was statistically significantly more retroverted, whereas D and H were not associated with dominance (p<0.01, p=0.9, and p=0.98). It was found that the glenoid sizes were very highly correlated with the patient's height, and it was highly correlated with the patient's weight (p<0.01 and p<0.01). On the other hand, height and weight were not correlated with the glenoid version (p=0.47 and p=0.81, respectively). There was no statistically significant relationship between BMI and glenoid sizes and glenoid version (p=0.14 and p=0.46, respectively).

Conclusions

Females in Turkish society had small glenoid sizes. Male gender, height, and weight were positively correlated with large glenoids. The glenoid was more retroverted on the dominant side. These findings should be considered in preoperative planning in Turkish society.

## Introduction

Reverse shoulder arthroplasty, whose principles underpin modern designs were defined by Paul Grammont, is becoming increasingly common [1]. One of the most important parameters determining treatment success and implant survival rates in reverse shoulder arthroplasty is placing the proper size implant in the optimal orientation [2]. This is possible with the use of a baseplate and glenosphere suitable for the patient's original glenoid [3]. However, glenoid morphology may show variation in different ethnic groups [4]. Therefore, it is important to know the glenoid morphology and the demographic parameters that this morphology may be associated with in preoperative planning in patients who will undergo reverse shoulder arthroplasty [5]. It is possible to determine this in the individual measurements to be performed in the preoperative evaluation for each patient. However, it is important to determine the mean glenoid size and glenoid version of the population in determining the prosthetic components that will need to be used more frequently in long-term planning. It has been detected that the data in the literature on this subject mostly belong to Western society [5]. Considering that the mean height of individuals in Turkish society is shorter than in Western society, it has been predicted that the glenoid would be smaller in Turkish society [6]. This situation leads to questioning the suitability of reverse shoulder prosthesis components designed for the Western society for the Turkish society.

For the stated reasons, it was aimed to determine to mean glenoid size and retroversion in Turkish society and to examine their relationship with the dominant side, gender, height, weight, and body mass index (BMI).

## Materials and methods

Study design and setting

This retrospective study was carried out in Istanbul Avcılar Murat Kölük State Hospital. Institutional review board approval was obtained for the study on November 25, 2020 (no: 21277189).

Inclusion and exclusion criteria

Inclusion criteria were having both shoulders CT scan during thorax CT imaging, and being older than 18 years of age. Patients with glenoid fracture, arthrosis, shoulder surgery, or deformity were excluded from the study.

Patients' demographics

Between 2019 and 2021, 102 shoulders of 51 patients (24 females/27 males, 51 left/51 right) were retrospectively evaluated. The mean age was 41.69 (range: 18-73) years (Table 1).

**Table 1:** Demographic data of patients whose bilateral shoulder CT scan images were evaluated. Min: minimum; max: maximum; n: number; SD: standard deviation

Variables	Mean ± SD	Min-max
Age (years)	41.69 ± 13.24	18-73
Sex, n		
Female	24	-
Male	27	-
Side, n		
Left	51	-
Right	51	-

Image acquisition

**Figure 1:**
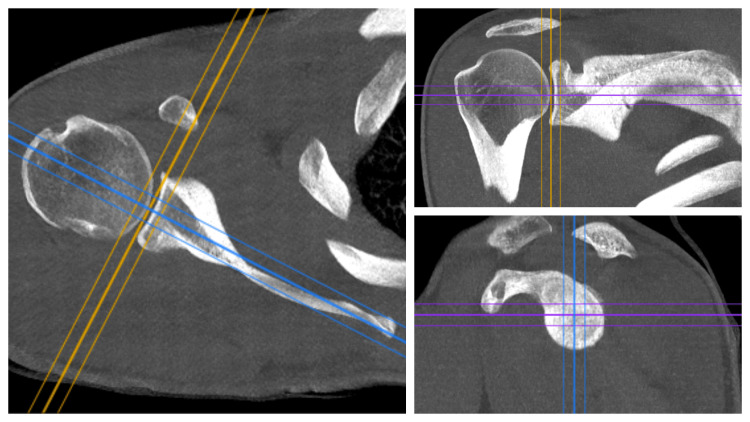
Standardized axial slices were obtained by reference to the line connecting the middle of the glenoid fossa surface in the coronal and sagittal planes and the medial pole of the scapular spine.

Computed tomography (CT) images were obtained using a 16‐slice CT scanner (Alexion; Otowara, Japan: Toshiba Medical Systems). Electronic images of the patients were downloaded from the image archiving and communication system (Ankara, Turkey: Extreme PACS) as DICOM files. The downloaded DICOM files were transferred into the imaging software RadiAnt DICOM Viewer (2021.2.2; Poznan, Poland: Medixant), and measurements were carried out on standardized images obtained with three-dimensional corrections (Figure 1).

Measurements

**Figure 2:**
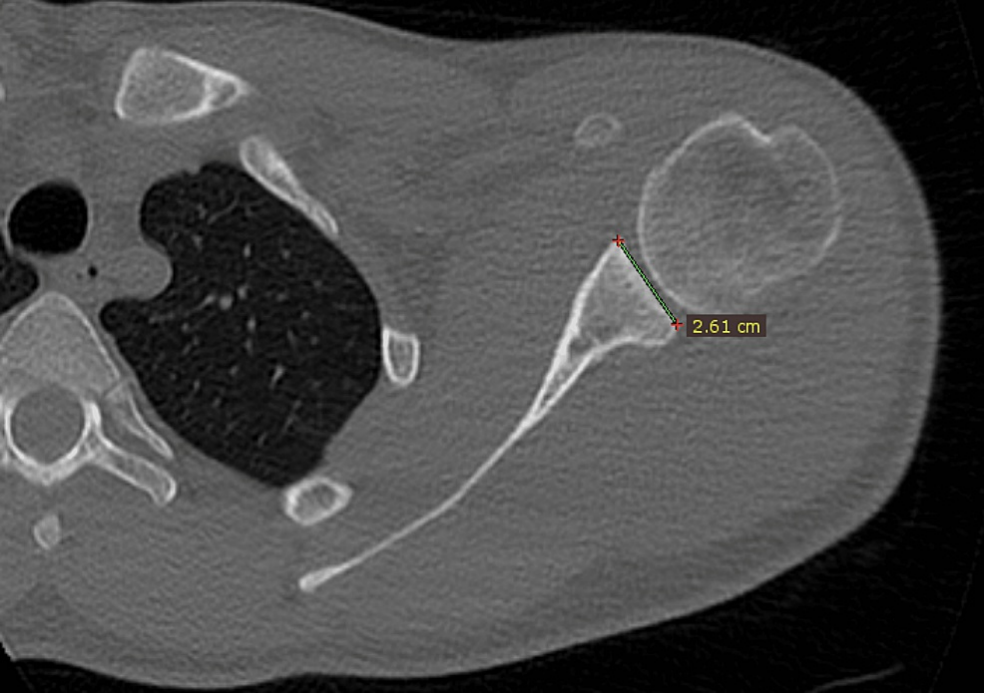
Measurement of the glenoid diameter in the anterior-posterior direction on the standardized axial slice.

**Figure 3:**
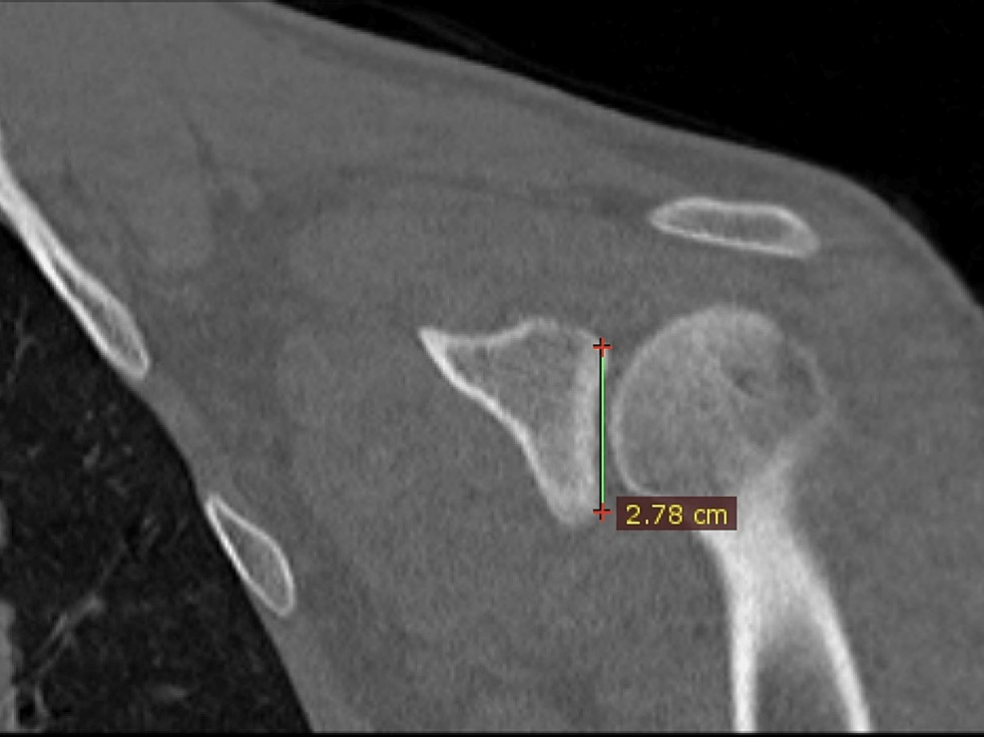
Measurement of the glenoid height in the superior-inferior direction on the coronal slice.

**Figure 4:**
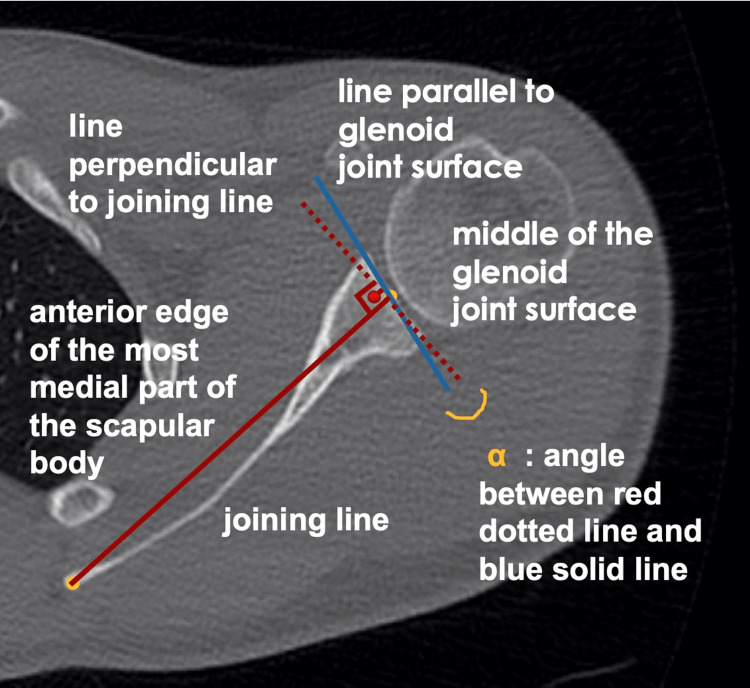
Measurement of the glenoid version with the Friedman method on the standardized axial slice. The line joining the middle of the glenoid joint face and the anterior edge of the most medial of the scapular body is used in the axial slice. The glenoid version is defined as the angle between this line and the line parallel to the glenoid fossa joint surface.

Measurements were carried out by an orthopedic surgeon experienced in shoulder and elbow surgery. Glenoid anterior-posterior diameter (D) and glenoid version (GV) were measured in axial slices, and glenoid height (H) was measured in coronal slices (Figures 2-4).

The correlation of these parameters with gender, height, weight, and dominant side was examined. The dominant side was determined by questioning which hand was primarily used in daily living activities.

Statistical analysis

Descriptive statistical methods (mean, standard deviation, minimum, maximum) were used to evaluate the data. Kolmogorov-Smirnov test was used to evaluate the distribution of the variables. The measured values were analyzed using the independent samples t-test and Pearson correlation coefficient at a 95% confidence interval. statistical analyses were performed using the SPSS version 26 (Armonk, NY: IBM Corp.).

## Results

The mean D was 25.79±4.44 mm, the mean H was 29.08±4.08 mm, and the mean GV was -0.99°±0.92°. The mean height of the patients was 162±16.23 cm and the mean weight was 71.9±15.36 kg (Table 2).

**Table 2:** Data of the patients' mean glenoid morphology measurements, height, and weight. Min: minimum; max: maximum; SD: standard deviation

Variables	Mean±SD	Min-max
Glenoid diameter (mm)	25.79±4.44	18-36.4
Glenoid height (mm)	29.08±4.08	19.9-40.2
Glenoid version (degree)	-0.99±0.92	-3.2-1.3
Patients height (cm)	162±16.23	132-194
Patients weight (kg)	71.9±15.36	44-110

The left side was dominant in 19 patients and the right side was dominant in 32 patients. The mean D on the dominant side was 25.8±4.47 mm, the mean H was 29.07±4.09 mm, and the mean GV was -1.66°±0.69°. The mean D on the non-dominant side was 25.78±4.45 mm, the mean H was 29.08±4.1 mm, mean GV was -0.32°±0.58° (p=0.9, p=0.98, and p<0.01, respectively). The mean D was 28.74±3.07 mm, the mean H was 31.3±3.89 mm, the mean GV was -1°±0.89° in males, whereas the mean D was 22.48±3.24 mm, the mean H was 26.58±2.57 mm, the mean GV was -0.98°±0.97° in females (p<0.01, p<0.01, and p=0.92, respectively) (Table 3).

**Table 3:** Comparision of glenoid morphology based on hand dominance and sex. *Independent samples t-test. Min: minimum; max: maximum; SD: standard deviation

Hand dominance	Dominant side		Non-dominant side		p-Value
	Mean±SD	Min-max	Mean±SD	Min-max	
Glenoid diameter (mm)	25.8±4.47	18.2-35.1	25.78±4.45	18-36.4	0.9*
Glenoid height (mm)	29.07±4.09	19.9-40.1	29.08±4.1	20-40.2	0.98*
Glenoid version (degree)	-0.32±0.58	-2.6-0.6	-0.32°±0.58°	-1.4-1.3	<0.01*
Sex	Male		Female		
Glenoid diameter (mm)	28.74±3.07	23.4-36.4	22.48±3.24	18-32.4	<0.01*
Glenoid height (mm)	31.3±3.89	19.9-40.2	26.58±2.57	22.4-30.4	<0.01*
Glenoid version (degree)	-1±0.89	-3.2-1.3	-0.98±0.97	-2.8-1.2	0.92*

Correlation coefficients for the relationship between height and D, H, and GV were r=0.83, r=0.76, r=0.07, respectively (p<0.01, p<0.01, p=0.47, respectively). Correlation coefficients for the relationship between weight and D, H, and GV were r= 0.7, r=0.66, r=0.02, respectively (p<0.01, p<0.01, p=0.81, respectively). Correlation coefficients for the relationship between BMI and D, H, and GV were r= -0.15, r= -0.11, r= -0.07, respectively (p=0.14, p=0.3, p=0.46; respectively) (Table 4).

**Table 4:** Correlations of glenoid morphology based on patient's height, weight, and BMI. *Pearson correlation analysis. BMI: body mass index

Variables	Height		Weight		BMI	
	r-value	p-value	r-value	p-value	r-value	p-value
Glenoid diameter (mm)	0.83	<0.01*	0.7	<0.01*	-0.15	0.14*
Glenoid height (mm)	0.76	<0.01*	0.66	<0.01*	-0.11	0.3*
Glenoid version (degree)	0.07	0.47*	0.02	0.81	-0.07	0.46*

## Discussion

It is known that the placement of the glenoid baseplate and glenosphere in the appropriate dimensions and in the correct orientation directly affects the stability and implant survival in reverse shoulder arthroplasty surgery [2,3]. This indicates the importance of preoperative planning and having prosthetic components of appropriate sizes available [2]. However, the majority of prostheses designed today have been prepared by prioritizing European and American patient populations [5]. Previously, Shimozono et al. drew attention to this topic and reported that the indicated designs may not be suitable for the Japanese patient population [5]. They reported that the mean glenoid width and height in the Japanese population were 28.1 ± 1.6 mm and 35.8 ± 2.2 mm in males and 23.4 ± 1.7 mm and 30.8 ± 1.8 mm in females, respectively [5]. In parallel with Shimozono et al., Mizuno et al. also pointed out that the minimum baseplate size is 25 mm in current prosthesis designs and reported that these designs may not be suitable for the Japanese patient population [7]. In that study, which also examined the French population, mean glenoid width and height were reported as 28.7 ± 2.1 mm and 37.3 ± 1.9 mm in men and 24.7 ± 1.7 mm and 33.5 ± 1.8 mm in women, respectively, in the French population [7]. Iannotti et al. reported that the mean glenoid width and height in non-Hispanic white Americans were 29 ± 3.1 mm and 39 ± 3.7 mm, respectively [8]. In our study, the mean glenoid anterior-posterior diameter of 28.74 ± 3.07 mm and glenoid height of 31.3 ± 3.89 mm in males, and the mean glenoid anteroposterior diameter of 22.48±3.24 mm and a glenoid height of 26.58±2.57 mm in females in the Turkish population were lower than the French patient population, non-Hispanic white Americans as well as Japanese patient population.

In our study, it was also investigated whether dominance has effects on glenoid sizes and glenoid versions. While it was seen that dominance did not affect the anteroposterior diameter and height of the glenoid, it was observed that the glenoid was more retroverted on the dominant side. In parallel with our findings, it was reported that glenoids were more retroverted on the dominant side in other studies [6,9]. The lack of dominance effect on glenoid anteroposterior diameter and height detected in our study was in line with the findings obtained in the study of Sarı et al. [6]. This finding supports that these data can be used in predicting glenoid size in patients who have previously contralateral shoulder CT scans obtained for other reasons, but may be misleading in estimating retroversion.

As previously reported in other societies, it was found in our study that the glenoid anterior-posterior diameter and height were higher in males than in females in the Turkish population [6-9]. However, it was observed that gender had no effect on the glenoid version similar to previously reported [10]. On the other hand, it has been reported in the literature that the glenoid is more retroverted in males in studies conducted on the American population [4,11]. Considering that the mean glenoid sizes in the Turkish society are smaller than the Western and Asian patient populations according to the data obtained in our study, it is of great importance to have the smallest size prosthetic components available preoperatively in female patients in the Turkish population.

The correlation of weight, height, and body mass index with glenoid size and version was also examined in our study. While the glenoid size was most strongly correlated with height, it was not correlated with body mass index. The correlation of glenoid size with the patient's height was consistent with the literature [4,6]. On the other hand, the glenoid version was not associated with weight, height, and body mass index in parallel with other studies [4,6].

Another study examining glenoid morphology in Turkish society was carried out by Sarı et al. [6]. The authors reported the mean glenoid width, height, and version to be 26.57 ± 3.02 mm, 31.8 ± 3.6 mm, and −0.93 ± 7.8°, respectively, in their study. Those values were 25.79±4.44 mm, 29.08±4.08 mm, and -0.99±0.92°, respectively, in our study. The notably large standard deviation of 7.8°, especially in the glenoid version obtained in their study, might be due to the lack of three-dimensional correction prior to measurement which is carried out in our study to exclude scapular tilt and inclination [12,13]. It was previously reported by Matsumura et al. that three-dimensional correction provides higher reliability and accuracy, especially in glenoid version measurements [14]. Another reported different finding from our study was that the glenoid size did not have a statistically significant difference in men and women.

Our study had several limitations with the main one being its retrospective nature. Measurements were carried out by a single observer once. Although the mean glenoid measurements in the Turkish society were obtained, the subjects were not patients with glenohumeral arthritis. Considering that the glenoid is more retroverted in patients with glenohumeral arthritis and the healthy bone stock is smaller due to erosion, measurements to be performed in patients with glenohumeral arthritis might be the subject of research for future studies [15,16]. Nevertheless, the determination of the mean values by evaluating the glenoid morphology for the first time with the three-dimensional correction technique in Turkish society constitutes a strong aspect of the study.

## Conclusions

Glenoid sizes are small in females in Turkish society. On the other hand, male gender, height, and weight are related to large glenoids. Sex is not related to the glenoid version, yet the dominant side is more retroverted. These findings should be considered in preoperative planning in Turkish society.
